# The Need for Pediatric Palliative Care in Romania: A Retrospective Study (2022–2023) Based on Quantitative Research and Analysis of Secondary Statistical Data

**DOI:** 10.3390/medicina61071282

**Published:** 2025-07-16

**Authors:** Mihaela Hizanu Dumitrache, Mădălina Duceac Covrig, Dana Elena Mîndru, Alina Plesea Condratovici, Geta Mitrea, Eva Maria Elkan, Antoanela Curici, Bogdan Gafton, Letiția Doina Duceac

**Affiliations:** 1 Faculty of Medicine and Pharmacy, “Dunărea de Jos” University of Galați, RO-800008 Galați, Romania; mh202@student.ugal.ro (M.H.D.); madalina.duceac@ugal.ro (M.D.C.); geta.mitrea@ugal.ro (G.M.); cojocarumariaeva@yahoo.com (E.M.E.); letimedr@yahoo.com (L.D.D.); 2 Department of Pediatrics, Faculty of Medicine, “Grigore T. Popa” University of Medicine and Pharmacy, RO-700115 Iasi, Romania; 3 Department of Cellular and Molecular Biology and Histology, “Carol Davila” University of Medicine and Pharmacy, RO-050474 Bucharest, Romania; antoanela.curici@umfcd.ro; 4 Department of Oncology, Faculty of Medicine, “Grigore T. Popa” University of Medicine and Pharmacy, RO-700115 Iasi, Romania; bogdan.gafton@umfiasi.ro

**Keywords:** palliative care, pediatrics, life-limiting diseases, medical resources, Romania, post-pandemic epidemiological needs, recovery

## Abstract

*Background and Objectives*: Estimating the need for palliative care for children is a crucial step in addressing the needs of children facing life-threatening conditions, while providing a powerful argument to combat unacceptably wide disparities in access to care. The need for palliative care for children in Romania remains insufficiently quantified. More accurate estimates are indispensable to assess the true extent of need and to support adequate policy responses. *Materials and Methods*: This retrospective cross-sectional study aimed to estimate the need for pediatric palliative care in Romania for 2022–2023. We analyzed secondary data obtained from the General Directorates of Social Assistance and Child Protection (DGASPC) in 41 counties and the six sectors of Bucharest. The analysis focused on life-limiting conditions as defined by the WHO Annex 3, Order no. 253/2018. *Results*: The study identified 14,499 pediatric cases with palliative care needs, showing a highly uneven national distribution, especially across diagnostic groups and age categories. We observed a higher number of cases in rural areas (7553) compared with urban areas (6946). Our own data do not include resource estimates; however, prior reports indicate only 50 palliative care beds for children in Romania. *Conclusions*: This study reveals a substantial and unevenly distributed need for pediatric palliative care in Romania, with notable disparities across age groups, diagnostic categories, and urban-rural areas. The identification of 14,499 eligible cases underscores the urgency of developing targeted policies and allocating adequate resources to ensure equitable access to specialized palliative services for all children in need.

## 1. Introduction

Pediatric palliative care is a specific key area of the healthcare system, aiming to improve the quality of life of children with incurable conditions through a holistic approach that includes medical, psychological, social, and spiritual support [1]. Globally, the World Health Organization (WHO) emphasizes the need to integrate palliative care services into all health systems, highlighting that millions of children require such services every year, but only a small proportion of them actually benefit from them [2]. According to a recent WHO report, approximately 21.5 million children globally require palliative care, 8 million of whom need specialized care, and the majority of whom live in low- and middle-income countries [1]. This highlights the importance of expanding pediatric palliative care services, especially in developing countries where resources are limited.

Although significant progress has been made internationally, palliative care services for children in Romania continue to be insufficient and unevenly distributed. While several studies, such as Pacurari et al. (2021) [3], have identified infrastructural deficits and staffing issues, none have provided national-level quantitative estimates to support targeted interventions or resource planning.

The data provided by the National Institute of Statistics [4] indicate a significant disparity between the need for pediatric palliative care and the available resources, highlighting the need for clear public policies for the development of this field. For example, in 2019, Romania had only 50 beds dedicated to children with life-limiting illnesses, a dramatically low number compared with the real needs [3]. This situation is aggravated by the fact that the majority of children in need of palliative care live in rural areas, where access to specialized medical services is extremely limited [4].

Therefore, the direct purpose of this study is to provide an accurate estimate of pediatric palliative care needs using national data.

Ultimately, the study aims to support improvements in quality of care and access through evidence-informed policy design.

## 2. Materials and Methods

### 2.1. Study Design

This study is a retrospective cross-sectional study based on data collected between October 2022 and December 2023. We extracted anonymized records from DGASPC branches in all counties and Bucharest.

The estimation of the number of children was performed by analyzing secondary data, processing information provided by DGASPC from 41 counties and the 6 sectors of Bucharest.

### 2.2. Participants

In this study, the term ‘children’ refers to all age groups from birth up to and including 18 years of age, thus covering newborns, infants, toddlers, school-age children, and adolescents:

- Ages 0 to 6;
- Aged between 7 and 13;
- Teenagers aged between 14 and 18.

We include children aged 0–18 years with a diagnosis matching the WHO classification of life-limiting or life-threatening conditions (WHO no. 253/2018, Annex 3) [5,6] (Table 1).

### 2.3. Data Analysis

- The dataset was processed using SPSS v.26.
- Descriptive statistics were calculated: total counts, means, standard deviations, and coefficients of variation (CV).
- The CV were used to assess relative dispersion across administrative units. In interpreting the CV, the following general criteria are used:
- CV < 15% → Low variability: the data are relatively homogeneous, with limited dispersion around the mean.
- 15% ≤ CV < 35% → Moderate variability: the data show a medium level of dispersion, indicating some differences between the analyzed units.
- CV ≥ 35% → High variability: the data are heterogeneous, with significant differences between units, which may suggest territorial inequalities, differences in access, reporting, or demographic structure.
- Due to the descriptive nature of the data and lack of individual-level outcomes, inferential tests (*p*-values, effect sizes) were not feasible. Future studies may incorporate hypothesis-driven statistical models.

### 2.4. Ethical Considerations

- The study used publicly available anonymized data provided by local institutions (DGASPC). No patient-level identifiers were included. As per national ethical guidelines for retrospective public data analysis, no approval from the research ethics committee was required.

We consider that we need to specify other aspects related to the content of the databases that we will analyze in order to understand exactly how we approached the palliative care issue in this research, the results obtained, and the limitations of our study. 

Please note that we have only requested information from the DGASPCs on the young population (0–18 years) because only this age group is of interest to us. Secondly, the databases contain information only about those patients who are in the records of the General Directorates of Social Assistance and Child Protection in Romania and who suffer only from a certain type of condition (those conditions for which we consider that palliative care services should be called).

## 3. Results

In the tables presented, several statistical measures are used to summarize the distribution of cases across the 47 DGASPC units, comprising 41 counties and 6 sectors. The variable N represents the total number of DGASPCs included in the analysis. Minimum and Maximum values indicate the lowest and highest number of children with a life-limiting or life-threatening illness reported in any single DGASPC, respectively. The *Sum* corresponds to the total number of such cases aggregated across all DGASPCs, amounting to 14,499 children. The Mean represents the average number of cases per DGASPC, calculated by dividing the total sum by the number of units.

Additionally, the Coefficient of Variation (CV) is reported as a measure of relative variability. CV is defined as the ratio of the standard deviation to the mean, expressed as a percentage, and provides insight into the degree of dispersion in the data relative to the average. A higher CV indicates greater variability or heterogeneity in the distribution of cases among DGASPC units, whereas a lower CV suggests a more uniform distribution.

This combination of descriptive statistics and variability measures offers a comprehensive understanding of the distribution and spread of cases across different administrative units.

Total Identified Cases: A total of 14,499 children aged 0–18 were recorded in the DGASPC system with conditions eligible for palliative care.

Analyzing the distribution by gender (Table 2), at the national level, the study highlights differences in numbers between girls and boys. For girls, the total number of patients is 6371, with a mean value of 135.55, a standard deviation of 53.74, and a coefficient of variability of 39.65%. These data suggest an uneven national distribution.

For boys, the total number of patients is 8128, with a mean value of 172.94, a standard deviation of 69.39, and a coefficient of variability of 40.13%. Also, in this case, the distribution is inhomogeneous.

Both distributions show moderate variability, indicating regional inequality.

Analyzing the distribution of patients by age groups (Table 3), a significant variability between categories is observed. For children aged 0–6 years, the total number of patients is 3456, with a mean value of 73.53. The standard deviation is 30.04, and the coefficient of variability is 40.85%, indicating an uneven distribution of cases in this age group.

For the category 7–13 years, the total number of patients reaches 6377 with a mean value of 135.68. The standard deviation is 54.47, and the coefficient of variability of 40.15% suggests a similar distribution is inhomogeneous.

For the 14–18 age category, the total number of patients is 4666, with a mean value of 99.28. The standard deviation of 47.15 and the coefficient of variability of 47.49% suggest an uneven distribution also in this case.

In total, most cases are recorded in the 7–13 age group (6377 patients), followed by the 14–18 age group (4666 patients) and the 0–6 age group (3456 patients).

The distribution of patients by residence (Table 4) reveals significant differences between rural and urban areas. In rural areas, the total number of patients is 7553, with a mean value of 160.7 and a standard deviation of 93.98. The coefficient of variability is 58.48%, suggesting an uneven distribution of cases.

In urban areas, the total number of patients is 6946, with a mean value of 147.79 and a standard deviation of 77.49. The coefficient of variability for urban areas is 52.43%, again indicating an uneven distribution of cases.

It is important to note that the number of patients in rural areas (7553) is higher than in urban areas (6946), which may suggest more limited access to care resources in urban areas, but also a need for additional resources in rural areas.

Regarding the distribution of patients according to diagnosis (Table 5), the following statistics were obtained:

- For group A (life-threatening diseases—curative treatment is possible but can fail) diagnosis, the total number of patients is 2004, with a mean value of 42.64, a standard deviation of 27.02, and a coefficient of variability of 63.36%, indicating a non-homogeneous distribution.
- For group B (life-limiting diseases—intensive treatment can prolong prognosis and improve quality of life) diagnoses, the total number of patients is 2203, with a mean value of 46.87, a standard deviation of 25.53, and a coefficient of variability of 54.46%, which again suggests an uneven distribution.
- For Group C (progressive diseases for which only palliative treatment is possible from onset), the total number of patients is 3469, with a mean value of 73.81, a standard deviation of 36.59, and a coefficient of variability of 49.57%, indicating an uneven distribution.
- For group D (non-progressive diseases causing constitutional fragility and high susceptibility to complications) diagnoses, the total number of patients is 6823, with a mean value of 145.17, a standard deviation of 67.74, and a coefficient of variability of 46.66%, which also suggests an uneven distribution.

It can thus be seen that the highest number of patients is recorded for a diagnosis in group D (6823), followed by group C (3469), group B (2203), and finally group A (2004). These data are illustrated in the following graph.

## 4. Discussion

Palliative care for children differs significantly from adult palliative care, requiring a specialized approach that includes not only physical treatments but also emotional and spiritual support for both the child and the family. This care starts with the diagnosis of the disease and continues throughout, regardless of whether curative treatments are given.

The results of the study highlight an uneven distribution of children with life-limiting conditions, as well as a major shortage of infrastructure and specialized staff. With only 50 dedicated beds at the national level, the capacity to provide adequate services is extremely low, highlighting a major crisis in the Romanian healthcare system [3]. This situation is in contrast with the recommendations of the World Health Organization (WHO), which emphasizes the importance of ensuring universal access to palliative care, especially for children with serious illnesses and their families [2]. According to a WHO report, approximately 21.5 million children globally require palliative care, and most of them live in low- and middle-income countries where resources are insufficient [1]. In Romania, this problem is aggravated by the lack of a coherent national strategy for pediatric palliative care, which leads to underutilization of available resources and low quality of services [3].

All children, regardless of income, illness, or age, should have access to nationally regulated palliative care services, including basic care [7,8]. In the context of the assessment of the number of children diagnosed with life-limiting illnesses in Romania, it is essential to emphasize the importance of a multidisciplinary approach in their care. Palliative care is not limited only to pain and symptom management but also includes essential aspects such as oral hygiene, which plays a crucial role in maintaining the child’s comfort and dignity. By increasing empathy and awareness among health care professionals, comprehensive care that meets the physical and emotional needs of the little patients can be provided [9].

Compared with the number of children with disabilities, according to official data [10] in June 2023, there were approximately 81,328 children with disabilities registered in Romania, only a small fraction of whom benefit from specialized medical services and adequate support. Compared with the 14,499 children diagnosed with life-limiting illnesses, we note that a significant proportion of this vulnerable population lacks access to essential palliative services. Approximately 17.83% of children with disabilities in Romania require not only palliative care but also recovery programs tailored to their specific needs. These personalized programs are essential to improve their quality of life, ensuring therapeutic interventions tailored to each child, constant monitoring, and ongoing support for their families. This percentage indicates a significant need for specialized services, highlighting the importance of developing an integrated policy for equitable access to care.

Regarding the distribution by age groups, the three age groups are characterized by certain particularities that both the medical staff and the palliative care providers must take into account: In the first age group (0–6 years) there are children who are still totally dependent on the family; they are at very high risk of not being included in the education system, and even if this is possible (the condition they suffer from allows school attendance), they may be to a greater extent neglected by the family (especially in very poor families with low level of education, families with many children, families living in rural especially isolated communities, etc.), not only because they are unable to contribute to the well-being of the family (by working to earn an income) but also because, through their illnesses, they are a very heavy drain on the family. The lack of interest in these children on the part of the family and some communities is also accentuated by the fact that adults do not see this as a loss—the child is too young—and, above all, that they anticipate the difficulties raised by the illness.

The risk that these children do not end up in the medical records, let alone the attention of palliative care services, is very high. This may also explain the lower share of this age group compared with the others.

The second age group (7–13 years) is children who, where possible, can be integrated educationally. Because of health problems and frequent hospitalizations, they may have a very high number of absences, which can eventually lead to dropping out of school. Attending school or any other place where they can come into contact with people of the same age or with adults who can integrate them is very important for the psycho-physical state of these children, who are at the age when they begin to realize that they are ‘different’.

We note that among the three age groups, this is also the most numerous. The explanation for the large number of children aged 7–13 years in the D.G.A.S.S.P.C.’s records may be the fact that they are somehow included in other records—school records, for example.

The next dominant group is the group of children in need of palliative care who fall into the 14–18 age group. In the medium and long term, this group raises another question: what will happen to them and their families when they become adults? This age group includes those children who are already aware of the problems facing them and their families and who are going through a rapid and painful process of maturing. The risk for this age group is that once they turn 18, they may no longer benefit from some of the facilities that are available to children, but which they still need.

In terms of residential environment, our analysis revealed that the majority of children live in rural areas, which means that there is a risk of worsening conditions, a decrease in quality of life, and an increased risk of death as poverty accelerates the deterioration of health. Access to palliative care services would partly reduce the impact of children’s living conditions on the course of their conditions. Through such services, children, especially the very poor, would benefit from care that the family is unable to provide due to lack of financial resources and education.

The discrepancies between rural and urban settings, where the majority of affected children live (7553 patients), access to specialized medical services is limited, and families face financial and logistical difficulties in obtaining the necessary care [4]. Studies show that in rural areas, the rate of access to specialized health services is up to 50% lower than in urban areas, leading to a worsening of children’s health and an increased risk of premature death [11].

Moreover, the predominance of rural patients suggests difficulties in access to specialized medical services. This situation is aggravated by the fact that many families in rural areas lack the financial resources to transport children to urban health centers, and the lack of education and awareness about palliative care leads to underdiagnosis and undertreatment of conditions [3]. Other studies show that rural children are more at risk of not receiving palliative care services, leading to a significant decrease in their quality of life and that of their families [1]. Lack of medical education among rural families also contributes to an underestimation of the severity of children’s conditions, leading to a delay in seeking medical help [4].

The distribution of patients according to diagnosis, analyzed in Table 5, should also be understood in terms of the classification of pathologies into clinical groups, each with distinct specificities in terms of disease course, therapeutic possibilities, and the need for palliative support. The classification used groups diseases into four categories (A-D), according to the progressive nature of the disease, whether curative options are available, and the associated level of clinical vulnerability.

Group A conditions include cancers, hematologic malignancies, and severe organ failure (cardiac, respiratory, renal, hepatic). The high variability (CV = 63.36%) observed in this category reflects the significant heterogeneity of the pathologies included, but also large differences in access to care and prognosis [12].

Group B includes conditions that, although not curable, can benefit from intensive treatments that help prolong life and improve quality of life. These include cystic fibrosis, HIV/AIDS, progressive muscular dystrophy, various inflammatory diseases, and chronic organ failure. Although these diagnoses are varied, their distribution among patients is less heterogeneous compared with group A, as indicated by a coefficient of variability of 54.46%. This value suggests a relative consistency in the way these conditions are diagnosed and treated, but also a possible stability in the allocation of care [13].

Group C refers to progressive, inevitably progressive diseases for which there are no curative treatment options from the outset. In these cases, palliative intervention is the only available option. This includes rare genetic diseases, metabolic storage disorders, or severe neurodegenerative diseases. Although the challenges are major, the distribution of these cases seems somewhat more even (CV = 49.57%), which may suggest better recognition and reporting of these conditions among health professionals, but also a more structured approach to their management.

Group D covers situations where conditions are not necessarily progressive but cause a constant and severe state of vulnerability. These are patients usually living with a major neurological disability—such as severe cerebral palsy, severe trauma to the central nervous system, or post-infectious sequelae. They are in constant need of complex care and are highly susceptible to complications. With the highest number of cases of all categories (6823), this group reflects a worrying but predictable reality, as these conditions imply a long life expectancy in conditions of total dependence on external support. The coefficient of variability of 46.66% confirms that while there is some uniformity in the patient profile, differences in access to care and individual needs remain significant.

This classification supports the interpretation of statistical differences, as each group implies different therapeutic approaches, medical resources, and support needs for the patient and family. Thus, not only clinical severity but also the nature of the disease and therapeutic perspective influence access to palliative care [14,15].

A large body of research emphasizes that relationships between family members and links with the community become extremely complex when children diagnosed with chronic illnesses or disabilities are involved in care. In such situations, family dynamics can undergo significant changes, with parents facing emotional stress and financial challenges, and siblings experiencing feelings of neglect or excessive responsibility. At the same time, social support, whether from relatives, friends, or the wider community, plays a key role in mitigating the impact of these difficulties. Studies show that coping strategies used by families—such as developing a support network, access to specialized services, and participation in support groups—contribute significantly to social integration and maintaining the emotional well-being of these children. In the absence of adequate social support and effective coping strategies, families may become isolated and exhausted, which directly affects the child’s health and development [16,17].

For example, a systematic review of the literature examined the effect of age on the associations between household income and general health status from birth through adolescence in different countries. The results show that, in many countries, income-related health inequalities persist or widen as children grow up. This suggests the need for early interventions to improve health equity across the life course [18].

A report by a non-governmental organization in Romania points out that massive absenteeism from school due to chronic diseases negatively influences children’s school performance and can be a demotivating factor for further studies, in the absence of curricular adaptation or finding a form of schooling adapted to the needs of the student. These findings emphasize the importance of early interventions and appropriate support to prevent school dropout among children with chronic conditions [19].

Concerns and numerous studies show that the transition from pediatric to adult palliative care is often significantly flawed, with only 23% of young people with chronic conditions receiving specialized services from childhood. The lack of well-structured transition programs and dedicated coordinators compounds these difficulties. Without adequate planning, adolescents and young people with life-limiting conditions remain deprived of adequate medical, psychological, and social support, and their families face insecurity and anxiety as their children lose their status as pediatric patients [20,21,22,23,24].

The older a person gets, especially if he or she is also suffering from a serious illness, the more support he or she needs, as well as the support the family needs. The public health system cannot fully provide the support needed by the family and the ill person, which is why access to palliative care services is very important, because palliative care is centered on the needs of the child and the family. Moreover, as the child grows older, so do the difficulties faced by the parents, who also grow older: medication, transportation, daily care, etc.

A study published in the Journal of Pain and Symptom Management [25] shows that about 150,000 people in Romania need palliative care every year. However, at the end of 2012, only about 6% of Romanian patients who needed such services had access to them. This discrepancy puts considerable pressure on family members, who become the main caregivers of patients in advanced stages of the disease [25]. Other scientific articles [13,14] emphasize that in Central and Eastern Europe, including Romania, there are significant gaps in end-of-life care and insufficient support for informal caregivers. This leads to an overburdening of family members, who have to manage alone the care of patients with advanced chronic diseases, often leading to burnout.

One of the most recent assessments of the development of pediatric palliative care globally [26], published in 2020, places Romania among the third category of countries with isolated palliative care services dedicated to children. The study concludes that only 21 of the 113 countries analyzed provide access to pediatric palliative care services at a reasonable level, and therefore, less than 10% of the world’s population under 20 years of age (35% of the global population) has access to timely palliative care services. More than 778 million children (30.7%), or about a third of the world’s children, live in the 55 countries (including Romania) where dedicated palliative care services are isolated and rare.

In 2019, in Romania, according to the latest annual report of the Romanian Association of Palliative Care, there were six palliative care services (continuous hospitalization) for children, two of which were hospice services developed by non-governmental organizations (30 beds), including three services with five beds each. According to the same document, there were only three non-governmental organizations with palliative care services for children with specialized teams at home [27].

In Romania, in recent years, there has been a growing effort to adequately integrate palliative care for children into the Romanian healthcare system. However, according to the study conducted by Pacurari et al. in 2021 [3], there are still significant barriers preventing the wider implementation of palliative care for children. These include a lack of qualified personnel in this field, insufficient financial resources, political instability in the country, and migration of qualified medical personnel, which remain significant barriers to the uniform development of these services nationwide [3].

In addition, the shortage of medical staff specialized in pediatric palliative care is another major problem. Professional training in this field is limited, and existing staff often find it difficult to meet the complex needs of children with terminal conditions [3]. This is in line with the findings of other international studies that emphasize the need to invest in continuing education and training for health professionals involved in pediatric palliative care [28]. For example, in countries such as the United Kingdom and the United States, specialized training programs for physicians and nurses in pediatric palliative care have led to a significant improvement in the quality of services provided [14]. In Romania, however, such programs are almost non-existent, leading to a lack of skills in the management of complex palliative care cases [3].

The results of this study underline the urgent need to expand pediatric palliative care services in Romania, with a focus on rural areas and disadvantaged regions. It is essential to allocate additional financial resources to develop medical infrastructure, increase the number of dedicated beds, and train specialized medical staff. It is also necessary to implement public policies to promote equitable access to palliative care for all children, regardless of region or background. These measures could include the creation of regional palliative care centers, the development of education programs for families and health professionals, and the promotion of partnerships between the public sector and non-governmental organizations to ensure wider coverage of pediatric palliative care services.

A comparison of the need for pediatric palliative care in Romania with other countries highlights a number of significant differences related to resources, infrastructure, and health policies. Analyzing the data from the Romanian study in comparison with other countries, it can be seen that, despite global progress, Romania is in a rather difficult situation in terms of pediatric palliative care [3,29,30,31,32].

This research highlights a critical situation in pediatric palliative care in Romania. The large number of children affected and the limited resources available underline the need for urgent reform in the healthcare system. Additional funds should be allocated to develop specialized services, increase the number of dedicated beds and facilities, and stimulate the training of medical staff. Through a coordinated and sustainable approach, equitable access to necessary palliative care for all children can be ensured.

Systemic challenges and solutions:

- Allocate additional funds for the development of pediatric palliative care infrastructure;
- Create strategies to ensure equitable access to palliative care services in rural areas;
- Closer collaboration between public authorities and non-governmental organizations to expand access to care;
- Support the training of medical staff specialized in pediatric palliative care.

It is clear that Romania faces a significant deficit in this area, but the proposed solutions could make an important difference in improving the quality of life of children with life-limiting diseases and supporting their families.

## 5. Limitations of the Study

It is likely that a significant number of pediatric cases remain unreported in official medical records due to factors such as geographic isolation, limited health literacy or engagement from caregivers, early age at symptom onset, parental minimization of symptoms, or ongoing diagnostic work-up. For this reason, we acknowledge the value of triangulating our findings with hospital-based or national health datasets in future studies to enhance data robustness and capture a more comprehensive picture of palliative care needs.

## 6. Conclusions

Access to pediatric palliative care is profoundly inadequate to current needs in Romania, manifesting itself in significant imbalances between urban and rural areas, geographical regions, age groups, and diagnostic categories.

The qualified human resources in the field of pediatric palliative care are insufficient, and their level of professional training is incomplete, lacking specific qualifications and continuous training adapted to the complex needs of this field.

### Strategic Recommendations

Integrate mandatory educational modules into the medical training curriculum and develop accredited postgraduate programs in pediatric palliative care to ensure adequate and up-to-date competencies for medical staff.

Improve integration of pediatric palliative care services into community and family medicine networks to enhance quality of life for children with life-limiting illnesses and their families by ensuring continuous and accessible support.

Develop a national strategy specifically for pediatric palliative care, distinct from adult palliative care, with clear and measurable objectives for implementation and service development.

This study’s findings highlight the importance of identifying high-need counties based on case distribution, in order to effectively allocate resources for palliative care.

Ensure adequate budgeting and implement a training plan with rigorous monitoring and control mechanisms to support the sustainable development of pediatric palliative care in Romania.

## Figures and Tables

**Table 1:** List of diagnoses eligible for palliative care.

	Health	Group
Life-threatening diseases—curative treatment is possible but can fail	Cancer, hematologic malignancies Organ failure (heart, respiratory, kidney, liver), severe congenital heart defects, etc.	A
Life-limiting diseases—intensive treatment can prolong prognosis and improve quality of life	Cystic fibrosis neuromuscular dystrophy Osteogenesis imperfecta form Severe congenital and disabilities HIV-AIDS Inflammatory diseases Panarteritis nodosa Crohn’s disease Kidney diseases—rapidly progressive glomerulonephritis, Toni–Debre–Fanconi syndrome, etc. Heart, kidney, liver failure.	B
Progressive diseases for which only palliative treatment is possible from onset	Chromosomal abnormalities Metabolic storage diseases, glycogenoses, mucopolysaccharidoses, some mucolipidoses, Gaucher’s B.Gaucher, Niemann–Pick’s B., etc. Hereditary degenerative diseases of the central nervous system—Werdning Hoffmann spinal muscular atrophy, Krabbe global leukodystrophy, Canavan spongiotic sclerosis, Alper polydystrophy, Leigh subacute necrotizing encephalomyelitis, etc. Acquired degenerative diseases—subacute sclerosing panencephalitis, multiple sclerosis, etc.	C
Non-progressive diseases causing constitutional fragility and high susceptibility to complications	Most are accompanied by severe neurological deficit—severe cerebral palsy with bed rest and multiple disabilities. Trauma to the central nervous system. Severe neurologic sequelae of central nervous system infections; meningomyelocele with severe neurologic damage.	D

**Table 2:** Gender distribution of patients.

Descriptive Statistics (at National Level)							
	N	Minimum	Maximum	Sum	Mean	Std. Deviation	CV
F	47	32	235	6371	135.55	53.744	39.65%
M	47	49	313	8128	172.94	69.393	40.13%
Valid N (listwise)	47						

**Table 3:** Distribution of patients by age groups.

Descriptive Statistics (Age Category)							
	N	Minimum	Maximum	Sum	Mean	Std. Deviation	CV
age_0_6_years	47	20	151	3456	73.53	30.036	40.85%
age_7_13_years	47	27	241	6377	135.68	54.471	40.15%
age_14_18_years	47	18	198	4666	99.28	47.149	47.49%
Valid N (listwise)	47						

**Table 4:** Distribution of patients by environment of residence.

Descriptive Statistics							
	N	Minimum	Maximum	Sum	Mean	Std. Deviation	CV
Rural	47	43	324	7553	160.70	93.975	58.48%
Urban	47	33	319	6946	147.79	77.487	52.43%
Valid N (listwise)	47						

**Table 5:** Distribution of patients by diagnostic category.

Descriptive Statistics (at National Level)							
	N	Minimum	Maximum	Sum	Mean	Std. Deviation	CV
A	47	0	109	2004	42.64	27.018	63.36%
B	47	6	109	2203	46.87	25.525	54.46%
C	47	9	174	3469	73.81	36.586	49.57%
D	47	23	313	6823	145.17	67.736	46.66%
Valid N (listwise)	47						

## Data Availability

All data from the first author and the corresponding author are available on request.
